# Urban land use influences microbial functional traits along a management intensity gradient

**DOI:** 10.3389/frmbi.2026.1946255

**Published:** 2026-09-30

**Authors:** Fadwa Khalfallah, Nicolas Legay, Sebastien Bonthoux, Amélie A. M. Cantarel, Jean-Christophe Clement, Arnaud Foulquier, Catherine Joulian, Xavier Le Roux, Agnès Richaume, Marie-Paule Norini, Jennifer Harris

**Affiliations:** 1 BRGM - French Geological Survey, Orléans, France; 2 Institut National des Sciences Appliquées Centre Val de Loire, Université de Tours, UMR 7324 Cités, Territoires, Environnement et Sociétés, Blois, France; 3 Long Terme Socio-Ecological Research, Zone Atelier Loire, France; 4 UMR 6554 Littoral Environnement Télédétection Géomatique (LETG), Rennes, France; 5 Université Lyon 1, CNRS, INRAE, Laboratoire d'Ecologie Microbienne (LEM), UMR 5557, UMR 1418 Villeurbanne, France; 6 Univ. Savoie Mont Blanc, INRAE, Centre Alpin de Recherche sur les Réseaux Trophiques et les Écosystèmes Limniques, Thonon-Les-Bains, France; 7 Univ. Grenoble Alpes, Univ. Savoie Mont Blanc, Centre National de la Recherche Scientifique, Laboratoire d'Ecologie Alpine, Grenoble, France

**Keywords:** biodiversity, land use, management intensity, microbial communities, urban green spaces, urban soil

## Abstract

**Introduction:**

Urbanization alters soil conditions and may strongly affect microbial communities, yet the functional consequences of these changes remain poorly understood. We investigated how urban land use and management intensity shape microbial diversity, functional traits, and interaction patterns in urban soils.

**Methods:**

We sampled 135 soils across eight land-use types in Blois (France), each assigned a management intensity level from 1 (low) to 4 (high). Bacterial and fungal communities were characterized using Illumina sequencing, fungal guilds were identified using FungalTraits, bacterial taxa were classified by life strategies, enzymatic activities were measured, and co-occurrence networks were constructed.

**Results:**

Land-use type and management intensity strongly influenced microbial composition, diversity, and functioning. Highly managed sites (Gardens, Sports fields) showed lower fungal 18S rRNA gene abundance, higher bacterial 16S rRNA gene copy numbers, reduced diversity, and lower nitrogen-related enzyme activities compared to less managed sites (Unused areas). These sites also exhibited more oligotroph-dominated and interconnected communities. Co-occurrence networks were less connected than those reported in semi-natural ecosystems, indicating more fragmented microbial associations in urban soils.

**Discussion:**

Our findings reveal that management intensity acts as a strong ecological filter shaping microbial functional traits and interaction patterns in urban soils. The reduced connectivity and functional shifts observed in intensively managed sites highlight the need to integrate microbial indicators into urban green-space planning and management.

## Graphical Abstract

**Figure:**
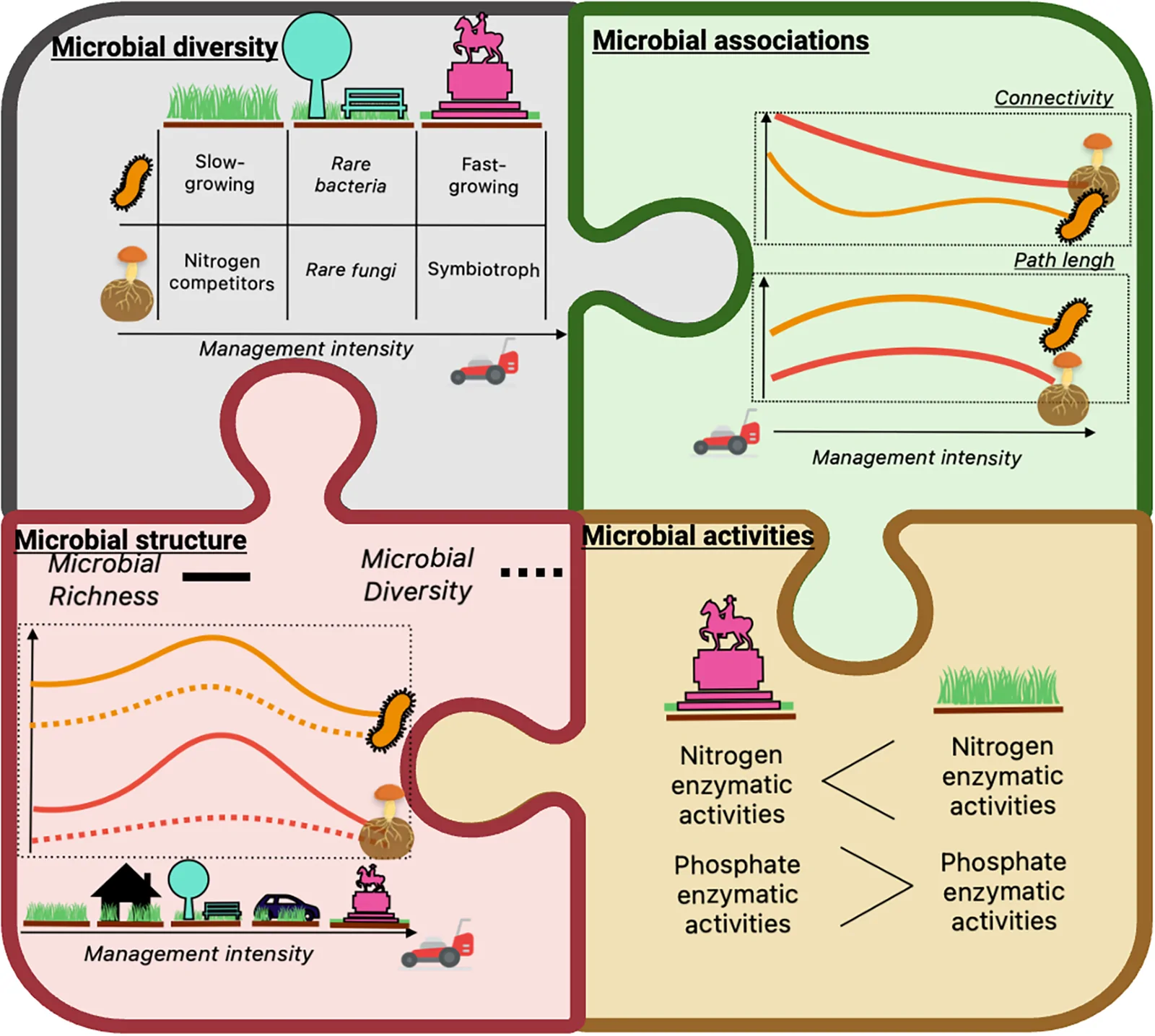

## 1 Introduction

Impacts of urbanization on biodiversity and ecosystem processes represents a key sustainability challenge in the 21st century (Fang et al., 2021). Between 2000 and 2018, over one million hectares were urbanized in Europe, which is more than eight times the amount of land that reverted to agriculture or nature (Evers et al., 2024). Compared to non-urban environments, urban soils are exposed to multiple, simultaneous disturbances such as construction, pollution, soil management practices, trampling and soil compaction. These selection pressures create heterogeneous microhabitats that may influence microbial community composition and increase within-site variability in community structure (Delgado-Baquerizo et al., 2018). Moreover, physical barriers in cities can lead to multiple habitats and their geographical dispersal (Pavao-Zuckerman and Coleman, 2007; Pickett et al., 2013; Ossola and Livesley, 2016). Additionally, land use (e.g., residential, recreative, industrial) and associated management habits such as fertilization, irrigation, plant selection and mowing frequency, further modify soil properties (Edmondson et al., 2014) and act as additional filters that shape microbial diversity and ecosystem functions (Joimel et al., 2016; Aronson et al., 2016; Fierer, 2017; Avolio et al., 2021).

Although previous work has demonstrated an effect of management intensity and land use in urban areas on microbial community composition and diversity (Baruch et al., 2020; Stephanou et al., 2021; Christel et al., 2023; Grierson et al., 2023), these studies remain seldom. A focus is generally made on specific aspects related to human health or extreme land use without considering the diversity of management practices within cities (i.e. agricultural gardens or industrial areas compared to natural parks). There is a lack of comprehensive data on how the composition, structure and functions of soil microbial communities respond to a variety of green space types of management in cities. This heterogeneity means that the diversity of urban soils and their microbial communities are often underexplored.

Microorganisms respond strongly to shifts in soil conditions and land use intensity, altering their community structure and activity, which makes them reliable indicators of soil health and human impacts (Ossola and Livesley, 2016; Philippot et al., 2024). Urban green space management practices are thus expected to affect microbial communities and consequently, soil functioning (Maron et al., 2011).

Urbanization can also filter microbial communities through life-history strategies, often leading to species homogenization and favoring generalist taxa adapted to disturbed and resource-variable environments (Aronson et al., 2016). For bacteria, these responses are frequently interpreted along the copiotrophic–oligotrophic continuum, analogous to the r/K framework (Maron et al., 2011; Ho et al., 2017; Stone et al., 2023). Copiotrophs are fast-growing bacteria that proliferate in nutrient-rich soils typical of intensively managed urban green spaces, whereas oligotrophs grow slowly, tolerate low nutrient availability, and contribute to the decomposition of recalcitrant organic matter in less intensively managed habitats (Trivedi et al., 2018). Shifts in the balance between these strategies therefore serve as indicators of land-use intensity and resource heterogeneity across urban environments (Karimi et al., 2018).

For fungi, functional traits and ecological guilds provide a complementary lens to understand community responses to urban pressures (Treseder and Lennon, 2015; Zanne et al., 2020). Saprotrophic fungi decompose complex organic matter (Maillard et al., 2023), mycorrhizal fungi form mutualistic associations with plants (Balestrini and Lumini, 2018; Brundrett and Tedersoo, 2020), and pathogenic or parasitic fungi exploit host organisms. Urban drivers such as nitrogen deposition or fertilization can shift guild composition—for example, by altering saprotrophic activity or favoring pathogenic taxa over mutualistic ones (Knorr et al., 2005; Zak et al., 2019; Lekberg et al., 2021). Habitat preferences also differ among guilds, with many ornamental and park plant species in cities predominantly associating with arbuscular mycorrhizal fungi (Smith and Read, 2010; Newbound et al., 2010). Consequently, changes in fungal guild representation provide functional indicators of management intensity, nutrient inputs and vegetation structure.

Together, these microbial life strategies constitute sensitive indicators of how urbanization and management practices filter microbial taxa, offering mechanistic insights into the reorganization of soil processes and ecosystem stability under urban pressures.

In this study, we aimed to determine how soil microbial communities respond to urban land-use types and to a gradient of management intensity in public herbaceous green spaces. Management intensity was graded by four levels, ranging from minimally managed areas (1–2 mowings per year) to high-intensity management (18–25 mowings per year, residue export, watering, scarification), with fertilizer inputs applied only in the highest category. This gradient captures the dominant forms of urban management that could modify soil resources, disturbance regimes and vegetation structure across the city. Specifically, we assessed how microbial richness, diversity, community structure and life-history strategies vary across land-use types and management intensities. We hypothesized that management intensity acts as an additional selective filter in urban soils, beyond the abiotic constraints imposed by land-use. Under high-intensity management—characterized by frequent mowing, fertilization, and intensive vegetation control—we expected (i) lower microbial richness and diversity, (ii) simplified community structure and reduced interactions, and (iii) a shift toward copiotrophic bacterial strategies adapted to nutrient-rich conditions. Conversely, in low-intensity or less disturbed areas, we anticipated (i) higher microbial diversity, (ii) more complex interaction networks, and (iii) a greater prevalence of oligotrophic bacterial and fungal guilds associated with stable or resource-limited environments.

Together, these hypotheses reflect the idea that microbial life strategies could serve as functional indicators of how urban land-use and management intensity filter soil communities and reshape ecosystem processes.

## 2 Materials and methods

### 2.1 Experimental site

Both the study site and the methods are described in detail in the data paper Norini et al., 2025. Briefly, soil sampling was carried out in the public space of the city of Blois (46,660 inhabitants in 2020), located in central France (47° 35′ 38″ N, 1° 19′ 41″ E). Mean annual temperature is 11.6 °C and annual rainfall is 640mm. The experimental design was developed in collaboration with the city’s management service to represent the entire urban territory and all relevant management intensities. Hence, we focused on herbaceous green spaces to avoid confounding effects from other vegetation types, except for the “Trees” condition, where samples were taken less than one meter from a tree and which consist of lines of trees associated to pedestrian paths. The sampling design targeted 135 sites which covered 8 different land uses across the city (**Figure 1**), and although unbalanced, it reflects the actual structure of the city since land use categories differ naturally in their spatial extent and frequency, and the distribution of samples therefore represents the real heterogeneity of Blois. We sampled 16 City Parks, 8 showcase Gardens, 10 Parklands, 33 Collective Residential areas (further referred to as “Residential”), 29 Roadsides (further referred to as “Road.co”), 5 Sports fields (further referred to as “Sport”), 7 Tree-covered areas (further referred to as “Trees”) and 27 Unused areas). City Parks are green spaces such as small parks and squares with an intermediate management intensity; showcase Gardens are highly maintained public gardens located in the historical old town; Parklands are large natural public green spaces; collective Residential areas are all green spaces associated to large social housing; Road.co are road components (road verges, roundabouts, traffic islands); Sports fields are highly modified soils with amendments and fertilizer inputs dedicated mainly to playing football and rugby; Unused areas are unused green areas with low management intensity reserved by the municipality for future buildings or flood absorption (**Table 1**). At each site, soil was collected within a clearly defined 40 m² quadrat positioned as close as possible to the center of the green space to avoid edge effects. Within this quadrat, nine soil cores (0–15 cm depth) were taken following a regular spatial pattern to capture local heterogeneity and homogenized into one composite sample. The sites were assigned into four management intensity categories ranging from: (1) corresponding to the lowest level of human influence, grass mowing 1–2 times per year; (2) corresponding to grass mowing 3–4 times per year (3) corresponding to grass mowing 10–15 times per year and (4) corresponding to the highest level of human influence, grass mowing 18–25 times per year with residue export, lawn watering and scarification. The management intensity gradient was established through a combination of practices, including fertilization, fungicide application, irrigation, soil management and mowing regimes. Fertilizers are applied exclusively to category 4 green spaces, while categories 1, 2, and 3 receive no fertilizer inputs. Together, these practices form a composite management profile rather than a strictly quantitative scale. For this reason, the 1–4 score should be interpreted as an ordinal gradient of overall management pressure, where each category reflects a distinct combination of human interventions rather than an equivalent stepwise increase in disturbance. 

**Figure 1:**
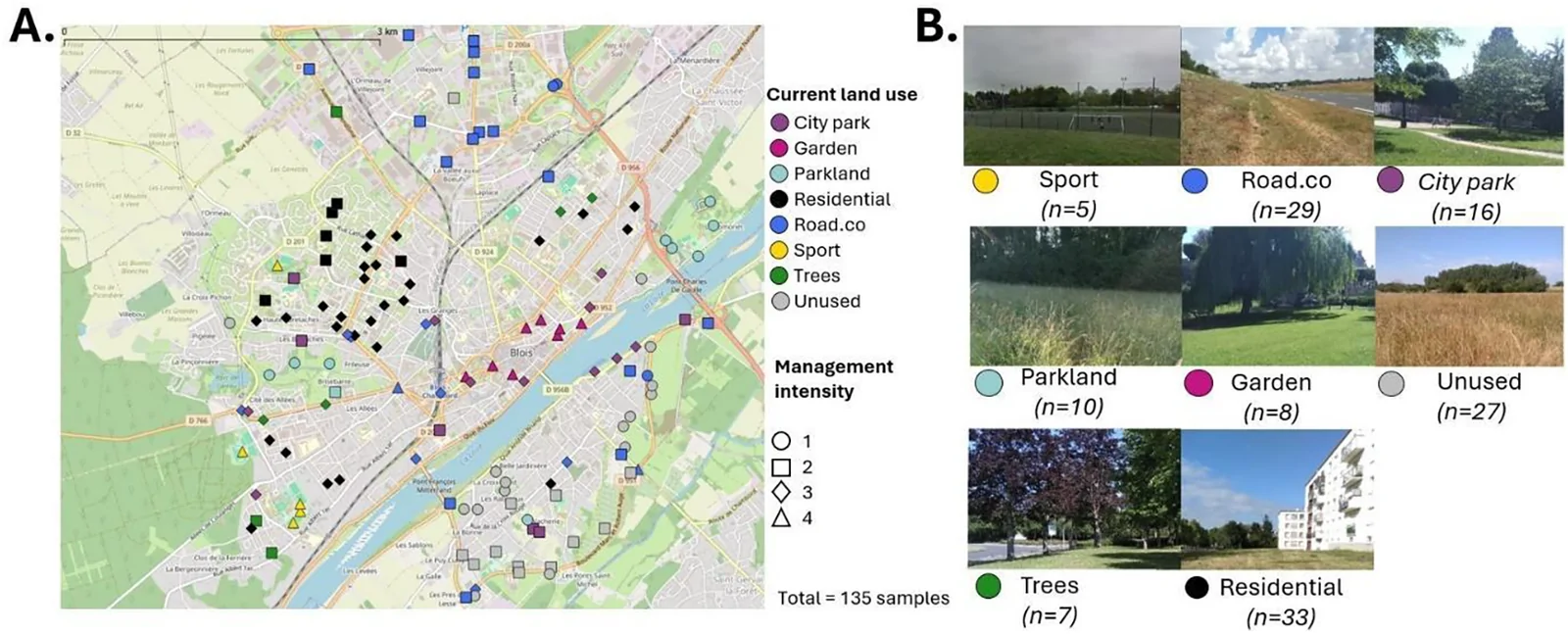
Localization **(A)** and land use **(B)** of the 135 sampled sites in Blois city (France).

**Table 1:** Urban land use categories, associated management-intensity levels, expected soil modification intensity (n = number of sites), and number of land use × management-intensity combinations (see **Supplementary Table SM1**).

Land use	Management intensity level	Soil modification level
Parkland	1	Low or moderate impact on soils
Unused	2-3	Low or moderate impact on soils
Road.co	1-4	Variable impact on soils
Trees	2-3	Moderate impact on soils
City park	2-3	Moderate impact on soils
Residential	3	Moderate impact on soils
Sport	4	Highly modified soils
Garden	4	Highly modified soils

Soil sampling was carried out in spring 2020 at 0–15 cm depth using a manual auger. At each sampling point, 9 soil cores were collected and homogenized to form one composite sample. The 135 fresh composite soil samples were sieved through a 2mm mesh and subsamples were stored at –20 °C for microbial related analysis (extracellular enzymatic activities, DNA extraction and quantification of 16S, 18S rRNA gene), and at 4 °C for the determination of soil moisture, soil texture, bulk density, total soil porosity, total carbon, total nitrogen and pH.

### 2.2 Soil physico-chemical properties

Gravimetric soil water content (SWC) was measured after drying subsamples at 105 °C for 48 hours. Bulk density was measured using a 250 cm³ soil core. Water holding capacity (WHC) was calculated after saturating soil cores with 100 mL of distilled water. Soil pH was measured in a 1:4 soil-to-water solution (Smith and Doran, 1997). Soil subsamples were air-dried, ground to <80 µm, and analyzed for total carbon (C) and nitrogen (N) content using a FlashEA 1112 elemental analyzer (Thermo Scientific, Flash EA 1112, Milan, Italy). Soil texture (sand, silt and clay fraction determination) was measured using a soil texture test kit (Model 1067; LaMotte, Chestertown, MD, USA) using the general method of mechanical analysis or soil separation.

### 2.3 Enzymatic activities

The potential activities of seven extracellular enzymes (α-Glucosidase (AG), β-1,4-Glucosidase (BG), β-D-cellobiosidase (CB), β-Xylosidase (XYL), leucine aminopeptidase (LAP), N-acetyl-β-glucosaminidase (NAG) and neutral phosphatase (PHOS)) were measured using standardized fluorimetric techniques (Bell et al., 2013). To ensure sample preservation prior to enzymatic analyses, all soil samples were frozen at –20 °C immediately after collection. This method allows to preserve enzyme activity patterns, and has shown that relative differences among treatments remain robust despite potential shifts in absolute activity (Lane et al., 2022).

### 2.4 MicroResp

Soil microbial respiration was measured using the MicroResp™ system (James Hutton Ltd., Aberdeen, UK). Both basal respiration (BR) and glucose-induced respiration (substrate-induced respiration, SIR) were measured.

### 2.5 DNA extraction and molecular analyses of soil microbial communities

DNA was extracted in duplicate from 0.5 g of fresh soil, using the FastDNA^®^ SPIN Kit for soil (MP Biomedicals, United States) per the manufacturer’s instructions with a FastPrep^®^ -24 instrument (MP Biomedicals, United States). The purified DNA was eluted in a buffer solution, quantified by the Quantifluor^®^ dsDNA sample kit fluorimetry and a Quantus Fluorometer (Promega, United States) following the manufacturer’s protocol and stored at −20 °C until further analysis.

The abundances of prokaryotic 16S rRNA genes (Bacteria + Archaea) and fungal marker (18S rRNA gene) were assessed by real-time quantitative PCR (qPCR) in a CFX Connect (BioRad) with primer sets 341F/515R (5’-CCTACGGGAGGCAGCAG-3’; 5’-ATTACCGCGGCTGCTGGCA-3’) for the bacterial abundance (López-Gutiérrez et al., 2004) and FR1/FF390 (5′-AICCATTCAATCGGTAIT-3′; 5′-CGATAACGAACGAGACCT-3′) for the fungal 18S rRNA gene copies (Prévost-Bouré et al., 2011). Data were expressed as gene copy numbers per gram of dry weight soil.

A portion of the 16S rRNA gene (V4-V5 region) was amplified using the barcoded, universal 16S primer set (515WF/918WR). A portion of the 18S rRNA gene was amplified using the universal 18S primer set (574WF/952WR; 5′-AICCATTCAATCGGTAIT-3′; 5′-CGATAACGAACGAGACCT-3′) (Hadziavdic et al., 2014). PCR reactions were performed using the AccuStart II PCR ToughMix kit, followed by cleaning (HighPrep PCR beads, Mokascience). Pooled triplicates were submitted for sequencing on Illumina MiSeq instrument at GeT-PlaGe (Auzeville, France). Amplicon libraries construction and Fastq generation were performed by INRAe Transfert (Narbonne, France).

### 2.6 Bioinformatics analysis

Bioinformatic processing of amplicon sequences was conducted using the FROGS workflow on the Galaxy platform, with full parameterization and step-by-step details provided in the **Supplementary Methods**. Shortly, bioinformatic analyses of the DNA sequences were performed using FROGS (i.e. Find Rapidly ASV with Galaxy Solution) (Escudié et al., 2018). Single-linkage clustering on sequences was performed using Swarm (with a read size of 250, aggregation distance clustering of 1) after merging, denoising and dereplication of Illumina sequencing with Vsearch. PCR chimeras were identified using VSEARCH, and both singletons and chimeras were subsequently removed from the dataset. ASVs were filtered based on several criteria, in particular the minimum ASV abundance as proportion or count defined to keep ASVs with at least 0.005% of all sequences. Bacterial ASVs was performed by BLAST against the SILVA 138.1 database (Quast et al., 2013). and Fungal OTUs were assigned using the UNITE Fungi 8.3 database (Abarenkov et al., 2010). The taxonomic affiliation of each ASV’s seed was assessed by BLAST and filtered with a minimum identity of 0.97 and a minimum coverage of 0.99. In total, we obtained 3 195–315 prokaryotic reads (normalized to 23,669 reads per sample; 2,413 ASVs) and 681–210 eukaryotic reads (normalized to 5,046 reads per sample; 1,679 ASVs). Archaeal ASVs (e.g., Crenarchaeota) were retained and included in all analyses of prokaryotic community composition.

### 2.7 Statistical analysis

All statistical analyses were conducted in RStudio (Team, 2020) using R version 4.1. Graphical representations were generated with ggplot2 (Wickham, 2016; version 3.4). Data manipulation and formatting were conducted using dplyr (Wickham et al., 2023; version 1.1.4) and tidyr (Wickham et al., 2024; version 1.3.1). Microbial diversity metrics (Shannon index and observed ASVs) were computed using the vegan package (Oksanen et al., 2024; version 2.6-8). Non-metric Multidimensional Scaling (NMDS) analyses were performed using vegan, and environmental fitting (envfit) was conducted using microeco (Liu et al., 2021; Oksanen, 2010). To evaluate potential causal pathways linking soil properties, enzymatic activities, microbial functional traits and microbial composition, we additionally performed mediation analyses using the mediate package (Tingley et al., 2014).

Microbial community matrices were first converted to relative abundances and subsequently Hellinger-transformed to stabilize variance and reduce the influence of highly dominant taxa. Bray–Curtis dissimilarities were computed from the transformed matrices and served as the basis for all beta-diversity analyses.

Because the experimental design was unbalanced and management intensity was not fully crossed with land use categories, the two factors could not be treated as independent. Management intensity was therefore considered a nested factor within land use. To account for this structure, permutation procedures in PERMANOVA were constrained within land use categories, ensuring that differences attributed to management intensity were evaluated only within the land use strata in which they occurred.

Differences in microbial community composition were assessed using PERMANOVA with 999 permutations. The model included land use and nested management intensity as explanatory variables, and Bray–Curtis dissimilarities as the response. Effect sizes (R²) were reported for each term to quantify the proportion of compositional variation explained. Because PERMANOVA can be sensitive to differences in multivariate dispersion among groups, homogeneity of dispersion was evaluated using betadisper followed by permutation tests.

Ordination analyses were conducted using non-metric multidimensional scaling (NMDS) based on Bray–Curtis distances. NMDS stress values were examined to ensure adequate representation of multivariate structure. Ellipses displayed in ordination plots represent data ellipses summarizing the distribution of samples within each land use category; they do not represent confidence intervals. Because ordination axes do not correspond to independent ecological gradients, differences among land use categories were interpreted as overlapping but statistically detectable shifts in community composition rather than discrete clusters. Principal coordinates analysis (PCoA) was used as a complementary visualization of major compositional gradients on the road.co condition.

Environmental variables—including soil physicochemical properties, enzyme activities, respiration rates, and gene-copy abundances—were fitted onto NMDS ordinations using the envfit procedure with 999 permutations. This method evaluates the strength of association between each variable and the ordination configuration independently. The management-intensity score (1–4) was treated as an ordinal categorical variable, reflecting distinct combinations of management practices rather than a continuous numeric gradient. Only variables showing significant associations with the ordination were retained for ecological interpretation.

Alpha-diversity metrics (richness, Shannon diversity, bacterial and fungal gene-copy abundances) were analyzed using linear or generalized linear models depending on distributional assumptions. Land use and nested management intensity were included as fixed effects. Model residuals were inspected to verify normality and homoscedasticity. Post-hoc comparisons were performed using Tukey’s HSD, and effect sizes (η² or R²) were reported to quantify the magnitude of differences.

To evaluate bacterial life-strategy patterns, taxa were classified as copiotrophs or oligotrophs based on published trait frameworks and curated databases. Assignments were performed at the lowest reliable taxonomic rank, using criteria derived from Stone et al. (2023); Ho et al. (2017), Fierer et al. (2007), and related literature. The complete mapping of all taxa, including the assigned strategy, taxonomic rank used, bibliographic source, and confidence level, is provided in **Supplementary Table 2**. Fungal guilds were annotated using Fungal trait database (Polme et al., 2020). Because the 18S marker provides limited taxonomic resolution compared to ITS, guild-level interpretations were restricted to high-confidence annotations. The taxa assignation for fungal guild and functional bacterial trait are provided from **Supplementary Tables SM3**–**SM6**.

To assess the effects of land use and management intensity, a Non-metric Multidimensional Scaling (NMDS) analysis was performed using the *vegan* package, based on the structure of bacterial and fungal community composition. Additionally, an *envfit* analysis was carried out using the *microeco* package to assess the impact of soil environmental variables on beta diversity.

Microbial co-occurrence networks were inferred using the NetCoMi package (Peschel et al., 2021; version 1.1.0). Before constructing the networks, we applied a standardized filtering procedure to ensure comparability across land-use categories. For each dataset, we first quantified the minimum observed ASV richness per sample, and this value was used as the richness threshold required for inclusion in the network analysis. The lowest richness observed among bacterial samples was 737 ASVs, while fungal samples showed a minimum richness of 206 ASVs. These thresholds ensured that all networks were constructed from samples with sufficient taxonomic resolution and avoided artefacts associated with low-richness samples. In addition to richness filtering, we applied a minimum read-depth criterion corresponding to the rarefaction depth used in the community analyses, namely 5040 reads for bacteria and 23,669 reads for fungi. Only samples meeting both criteria were retained for network inference. To maintain comparable sample sizes across networks, land-use categories were grouped when necessary. Garden, City Park and Sportwere combined into a “Management” group, while Parkland and Trees were combined into a “Tree-Park” group. This grouping allowed us to construct networks with similar numbers of samples and ensured robust comparisons between land-use types.

Networks were inferred using CLR-transformed ASV abundance data, Pearson correlations to estimate associations, and the pseudo-count method for zero handling. Community detection was performed using the fast-greedy clustering algorithm implemented in NetCoMi.

The urban-land use microbial networks were compared to the semi-natural networks (i.e. forest, scrubland, grassland), using the network properties of previous studies on bacterial and/or fungal communities: Jiao et al. (2016); Wang (2022); Nakayama et al. (2019); Chen et al. (2019b); Wu et al. (2021); Xue et al. (2022); Yu et al. (2022); Gui et al. (2023) and Wang et al. (2023). For each network we calculated the Average dissimilarity, a network property, that reflects compositional variation among samples and does not measure microbial interactions (Tang et al., 2021). We also computed the Average path length which measures the mean shortest distance between all pairs of nodes, reflecting the overall connectivity and potential interaction efficiency within the microbial network (Newman, 2003). We inferred the clustering coefficient that describes how much a node is connected with its neighbors, reflecting the local transitivity (Deng et al., 2012), and the Modularity describes the extent to which a network can be partitioned into topological modules, that is, groups of taxa that are more densely connected to each other than to the rest of the network. These modules represent structural communities within the network, but they should not be interpreted as functional units unless independent functional evidence is available (Deng et al., 2012). Finally, we calculated the Connectivity that represents the connection strength between nodes and serves as a measure to summarize hierarchical structures (Dai et al., 2019).

Extracellular enzyme activities were categorized based on their involvement in key biogeochemical cycles: enzymes associated with the carbon cycle (EEC = AG + BG + CB + XYL), those linked to the nitrogen cycle (EEN = LAP + NAG), and those involved in the phosphorus cycle (EEP = PHOS). Ratios of extracellular enzyme activities were calculated to represent microbial resource limitation associated with trade-offs between C, N and P acquisition: EEC: EEN and EEN: EEP ratios were calculated to represent the balance of C versus N, and N versus P acquisition respectively (Sinsabaugh et al., 2009).

## 3 Results

### 3.1 Microbial structure

#### 3.1.1 Impacts of soil use and management intensity on ASV richness and diversity

Microbial richness (number of ASVs) was significantly influenced by management intensity and current land use (**Supplementary Tables S1**, **S2**). Bacterial (972 and 1142 ASV) and fungal richness (322 and 508 ASV) were significantly higher under management intensity level 3 compared to levels 1 and 4 (and level 2 for fungi). For bacteria, this corresponded to a higher richness in City Park, Residential and Road.co compared to Unused. Fungal richness showed a similar pattern, though it was higher only in Residential compared to Unused and Sports. The Sports category had the lowest fungal richness compared to the other land uses, except for the Unused. Similarly, bacterial diversity (Shannon index) was higher under management intensity level 3 and lower under high (level 4) and low (level 1) management intensities. In terms of land use, microbial diversity was lower in the Sports compared to the Garden, Road.co and Unused land uses. For bacterial diversity, Sports and Unused areas had the lowest diversity compared to the Residential areas.

#### 3.1.2 Microbial community structure was influenced by site management and/or soil properties

Non-metric multidimensional scaling (NMDS) based on Bray–Curtis dissimilarities revealed clear differences in both bacterial and fungal community composition among land use categories (**Figure 2**). For bacteria the NMDS solution showed acceptable stress (stress = 0.15) and PERMANOVA indicated a significant effect of land use on community structure (adonis2, Bray–Curtis, R^2^ = 0.13, p<0.001; 7 df for land use). Tests of multivariate dispersion (betadisper and permutest) confirmed significant differences in dispersion among land use groups for bacteria (p=0.001) indicating that both location and spread contribute to beta-diversity patterns.

**Figure 2:**
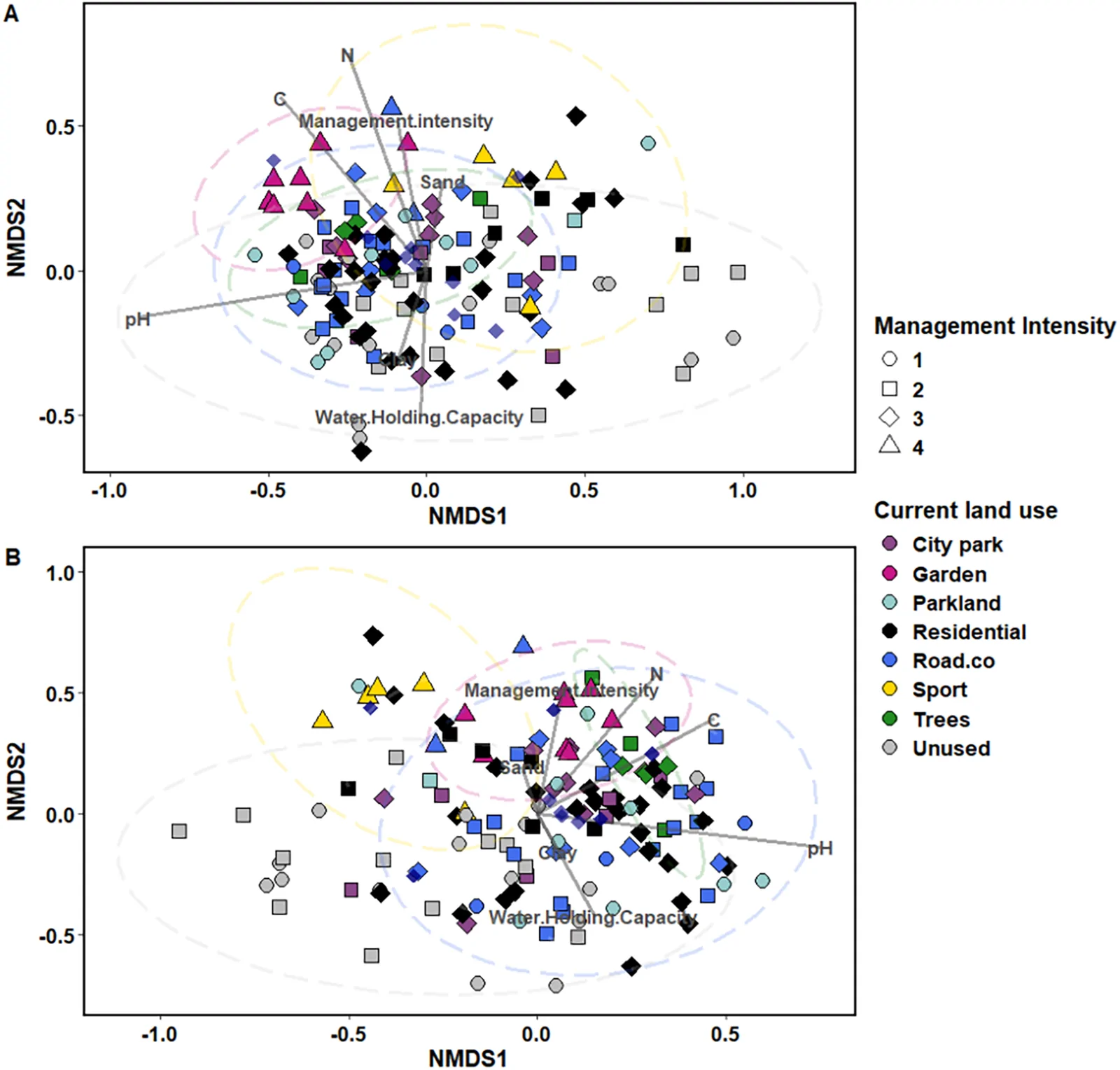
Non-metric multidimensional scaling (NMDS) plots illustrating how microbial community composition is related to soil variables and land use for **(A)** Bacterial communities **(B)** Fungal communities. Points represent sampling sites, color-coded by current land use (e.g., City Park, Garden, Parkland, Residential, Road.co, Sport, Trees and Unused) and shaped according to land use intensity (Management Intensity 1–4). Arrows correspond to soil variables (e.g., pH, nitrogen (N), carbon (C), sand content, water holding capacity, and management intensity), with arrow length scaled to the strength of the correlation as determined by environmental fitting (*envfit*). Ellipses represent 95% confidence intervals for groups based on land use categories.

For fungi, NMDS based on Bray–Curtis dissimilarities also showed distinct clustering of communities by land use, with low stress (stress = 0.13). PERMANOVA including both land use and management intensity, with permutations restricted within land use strata, revealed a significant combined effect on fungal community composition (adonis2, Bray–Curtis, R2 = 0.17, p=0.003; 8 df for land use). As for bacteria, multivariate dispersion differed significantly among land use groups (p=0.001), suggesting that fungal beta-diversity reflects variations in both group centroids and dispersion.

Overall, Garden, Sport, Road.co, Trees and Unused areas formed distinguishable clusters in ordination space for both bacteria and fungi, although some overlap remained (R2 = 0.11, p<0.001 for bacteria and R2 = 0.13, p<0.001 for fungi). When management intensity was considered within land use strata, fungal communities showed a clearer response to the combined effects of land use and management intensity than bacterial communities, for which the stratified PERMANOVA model was only marginally significant (p=0.101). These results support the conclusion that land use is a primary driver of microbial beta-diversity in urban soils, with management intensity exerting an additional, but more subtle, influence—particularly for fungi.

Significant differences between City Park and the other land uses were found based on physico-chemical properties (**Supplementary Table S3**). Specifically, soils in Parkland and Trees areas contained significantly less carbon and nitrogen than in City Park. Soils in Road.co were characterized by lower pH compared to soils in Residential, Sports and City Park areas. Additionally, sand contents were higher in City Park and Parkland soils, whereas they were lower in Sports and Residential areas.

Fitting of environmental variables with bacterial and fungal beta diversity (i.e. envfit analysis) revealed significant link between soil parameters and microbial community structure for most of the variables (**Supplementary Table S4**). The strongest relations were observed for soil pH (bacteria: R^2^ = 0.48, p=0.001; fungi: R^2^ = 0.44, p=0.001), total soil nitrogen (bacteria: R^2^ = 0.35, p=0.001; fungi: R^2^ = 0.34, p=0.001), total soil carbon (bacteria: R^2^ = 0.32, p=0.001; fungi: R^2^ = 0.28, p=0.001), and management intensity (bacteria: R^2^ = 0.16, p=0.001; fungi: R^2^ = 0.21, p=0.001). Indeed, for both bacteria and fungi, soil pH was associated with clustering of Road.co and Sports areas, while total soil carbon and nitrogen contents were associated with clustering of Garden and Unused areas (**Figure 2**).

### 3.2 Influence of land use on microbial community composition and life strategies

#### 3.2.1 Bacterial communities

The dominant bacterial phyla were Proteobacteria (24%), Actinobacteria (21%), Bacteroidota (19%), and Acidobacteria (16%) (**Figure 3A**). Actinobacteria showed a significantly higher relative abundance in Unused areas compared to Residential, Sport, and Road.co areas (**Supplementary Table S5**). Similarly, Actinobacteria had a higher relative abundance in management intensity level 1 compared to the highest levels (3 and 4) (**Supplementary Table S6**). In contrast, Bacteroidota had the lowest abundance in Unused areas. In terms of management intensity, their abundance was the lowest in level 1 compared to level 3. Proteobacteria abundances were higher in Residential than in Road.co areas (**Supplementary Table S5**). Chloroflexi showed higher relative abundances for the highest management intensity (level 4) compared to the others (**Supplementary Table S6**). In terms of functional groups, oligotrophic bacteria were more abundant in Unused compared to other land uses, except for Garden (**Figure 3B**; **Supplementary Figure S2**). In contrast, oligotrophic bacteria were less abundant in Residential compared to other land uses.

**Figure 3:**
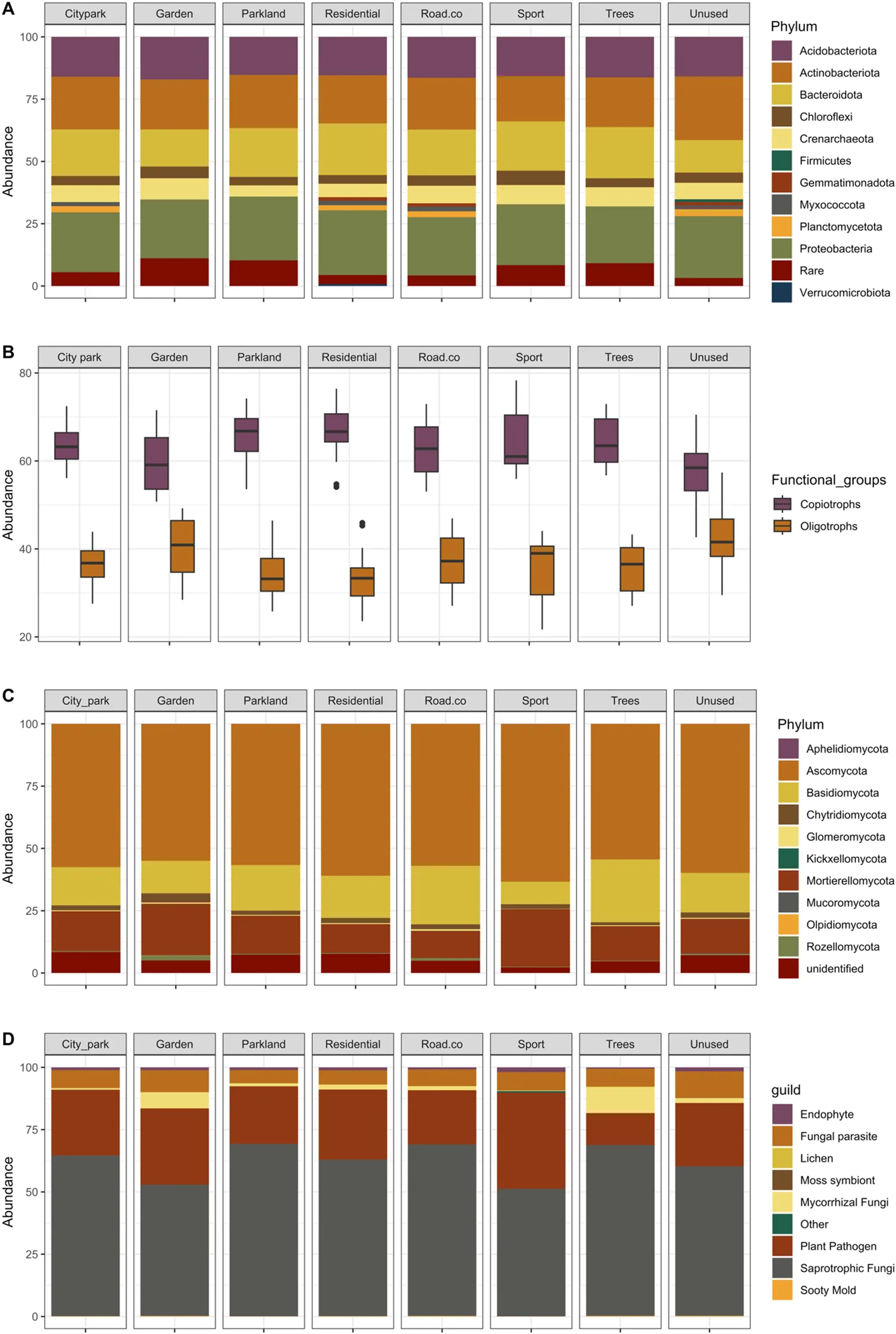
Relative abundances of microbial taxa and functional groups across different land uses. **(A)** Relative abundance of bacterial phyla, showing the distribution of major bacterial taxa (Rare represents taxa with less than 0.5% of the abundance). **(B)** Proportion of bacterial functional groups classified as oligotrophic or copiotrophic bacteria. **(C)** Relative abundances of fungal phyla. **(D)** Distribution of fungal guilds based on ecological functions. Each bar represents the mean relative abundance within a specific land use category.

Significant differences in relative abundance between land uses were identified at the order, family, class and genus level (**Supplementary Table SX**; **Supplementary Figure S3**). For example, *Solirubrobacter* and members of the *Gaiellales* order showed higher abundances in Unused soils compared to more intensively managed land-uses. In contrast, families such as *Microscillaceae* and *Chitinophagaceae* displayed lower abundances in Unused areas and were more prevalent in managed environments. Overall, these lower-rank patterns reveal clear land-use structuring that was not detectable at the phylum level.

#### 3.2.2 Fungal communities

The primary fungal phyla identified were Ascomycota (58%), Basidiomycota (17%), and Mortierella (16%) (**Figure 3C**). There was little variation in these major phyla across land uses (**Supplementary Table S5**). Basidiomycota showed lower relative abundances for the highest management intensity (level 4) (**Supplementary Table S6**). In contrast, Mortierollomycota had a higher relative abundance in the highest management intensity in comparison to levels 2 and 3. Differences between land uses were mainly observed for rare taxa, with Chytridiomycota (<1.8%) and Rozellomycota (<0.7%) showing higher relative abundances in Garden areas and for the highest management intensity (level 4). Aphelidiomycota (<0.4%) exhibited a higher relative abundance in the highest management intensity level compared to the lowest, and Mucoromycota (<0.3%) were more abundant in Unused areas.

Overall, saprotrophic fungi represented the dominant guild in urban areas (62%), followed by plant pathogens (25%) (**Figure 3D**). These fungal lifestyles varied according to land use, with plant pathogens being more prevalent in Residential and compared to Road.co areas. Mycorrhizal fungi were associated with Trees areas and showed the greatest variation for the fungal guild across land uses. Variations of mycorrhizal fungi were mostly explained by the dominant fungal tree trait at each sampling site (**Supplementary Figure S4**). Indeed, the variation of mycorrhizal fungi was mostly explained by tree-fungi associations (whether it was with AM fungi or ECM fungi) and not by land use. Similarly, the proportion of plant pathogens was influenced by tree-fungi associations, but also by land use. For saprophytic fungi, soil N concentration and pH explained most of the variations (**Supplementary Figure S4**).

Additionally, lichen showed the lowest relative abundance in areas with the highest management intensity while parasites (plant, animal, fungal) had the highest relative abundance within the highest management intensity (**Supplementary Table S6**). 

### 3.3 Soil enzyme activities and mediation pathways

Garden areas exhibited significantly lower EEC: EEN ratios compared to Residential, Sport, Unusedand City Park. Garden had the highest EEN: EEP ratio compared to other land uses except Road.co, Trees and Parkland areas (**Figure 4**).

**Figure 4:**
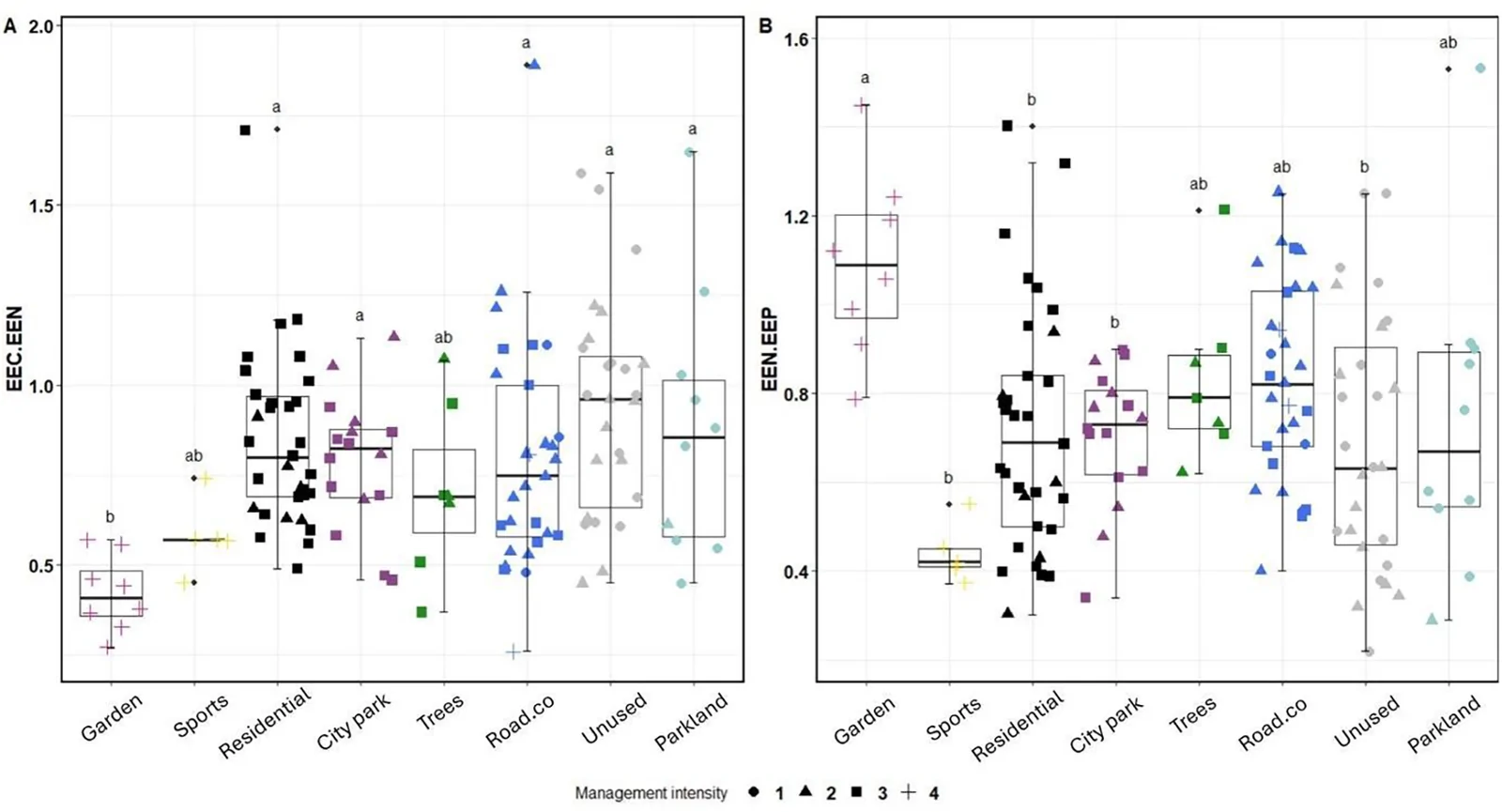
Enzyme activity ratios (EEC: EEN and EEN: EEP) across different land uses with **(A)** Ratio EEC:EEN, **(B)** Ratio EEN:EEP. The points represent sampling sites, color-coded by land use (e.g., City Park, Garden, Parkland, Residential, Road.co, Sport, Trees and Unused). Letters represent pairwise Tukey's comparison test (P<0.05).

Several dominant taxa showed significant indirect effects on carbon- and nitrogen-related enzyme activities. In particular, copiotrophic Proteobacteria partially mediated the relationship between β-glucosidase activity and both soil carbon and nitrogen contents (**Supplementary Table S8**). Actinobacteria significantly mediated carbon-acquisition enzyme ratios (EEC: EEN). For fungi, root endophytes emerged as the main fungal mediators linking carbon- and nitrogen-acquisition enzymes to soil C and N contents (**Supplementary Table S9**). Moss-associated symbionts also mediated several carbon-cycle enzyme pathways. In contrast, animal-associated fungal guilds mediated negative effects for a few enzyme–soil relationships.

### 3.4 Microbial associations

Microbial network properties differed according to land uses and management intensities (**Figure 5**; **Supplementary Table S10**). Bacterial network topology differed markedly among land use categories. Residential and Unused soils exhibited the largest connected components (relative LCC size = 0.93 and 0.96). In contrast, Parkland, Tree-Park, and Road.co networks were substantially more fragmented, with relative LCC sizes ranging from 0.53 to 0.56. Clustering coefficients were generally higher in less managed or structurally heterogeneous environments (Unused = 0.32; Tree-Park = 0.38; Management = 0.36), suggesting stronger local grouping of taxa. Modularity was highest in Tree-Park (0.61) and Management (0.62), whereas Residential and Unused networks displayed lower modularity (0.25–0.29). Positive edge percentages ranged from 70–72% in Residential and Unused soils to over 90% in Tree-Park, suggesting that highly structured or less disturbed environments support predominantly positive associations. Edge density was highest in Unused soils (0.015), reflecting a greater number of associations per taxon, and lowest in Road.co and Parkland networks (0.002–0.005), consistent with their fragmented structure. Natural connectivity was highest in Unused soils (0.029) and lowest in Road.co (0.006–0.009). Average path length was shortest in Unused soils (1.66) and longest in Tree-Park and Road.co (2.50–2.53).

**Figure 5:**
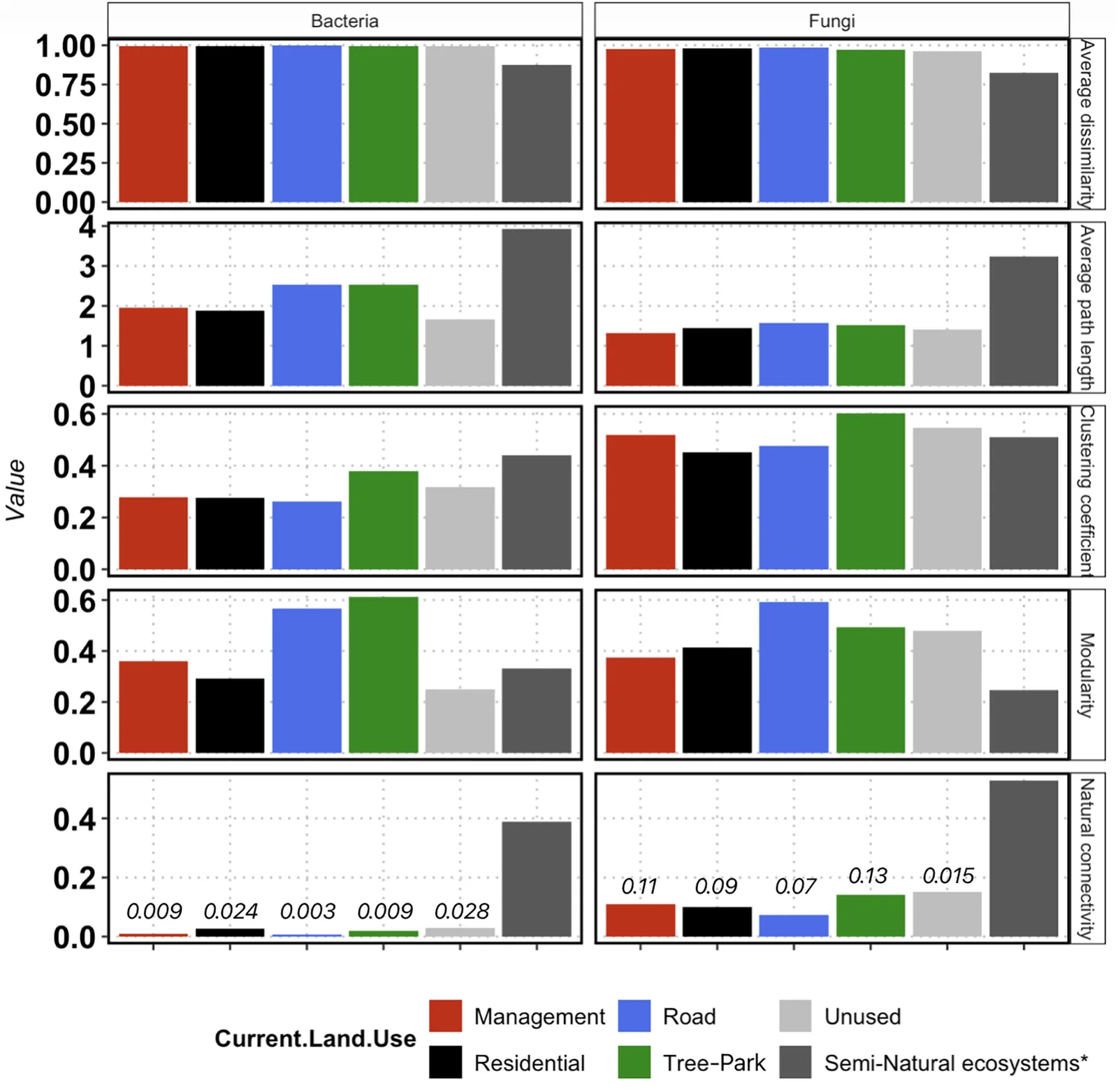
Microbial network properties according to different land use categories (Management, Road.co, Residential, Unused, and Tree-Park). “Management” includes Garden, City Park and Sport, while “Tree-Park” includes Parkland and Trees. “Semi-Natural Ecosystems” corresponds to the average microbial network properties observed in semi-natural ecosystems, including grasslands, forests, and shrublands based on a literature review (see Methods).

Fungal networks showed consistently higher levels of connection across land uses. Component size distributions typically consisted of one dominant connected component and a small number of minor ones. In the tree network, clustering coefficients were high (0.60), and modularity reached 0.49. Positive edge percentages exceeded 97%, and edge density (0.0336) and natural connectivity (0.1416) were markedly higher than in bacterial networks. Similar patterns were observed in other fungal networks, where LCC sizes exceeded 0.98 and the number of components remained low (5–26).

For both kingdoms the highest connectivity was displayed in the Unused areas and the lowest connectivity in the Road.co areas. The comparison between urban and non-urban networks, using average values of network properties from semi-natural ecosystems (forests, grasslands and shrublands), highlighted relatively low values of natural connectivity and average path lengths in the urban microbial community’s networks. However, the urban microbial communities displayed higher values of dissimilarity compared to the semi-natural networks.

### 3.5 A comparative approach

Multidimensional analysis of the functional and microbial parameters exhibited an effect of land use and management intensity on soil microbial and abiotic variables (**Figure 6**). ANOSIM analysis further confirmed a separation of land use (p<0.001) and management intensity (p<0.001). PCA analysis indicates that enzyme activities drive the first principal component in opposition to soil respiration, environmental DNA, bacteria/fungi ratio and the proportion of oligotrophic bacteria which were correlated to carbon and nitrogen. The second principal component is driven by fungal and bacterial richness and diversity. An envfit analysis revealed the impact of management intensity, carbon and nitrogen content, and water-holding capacity on functional microbial parameters (**Supplementary Table S11**). 

**Figure 6:**
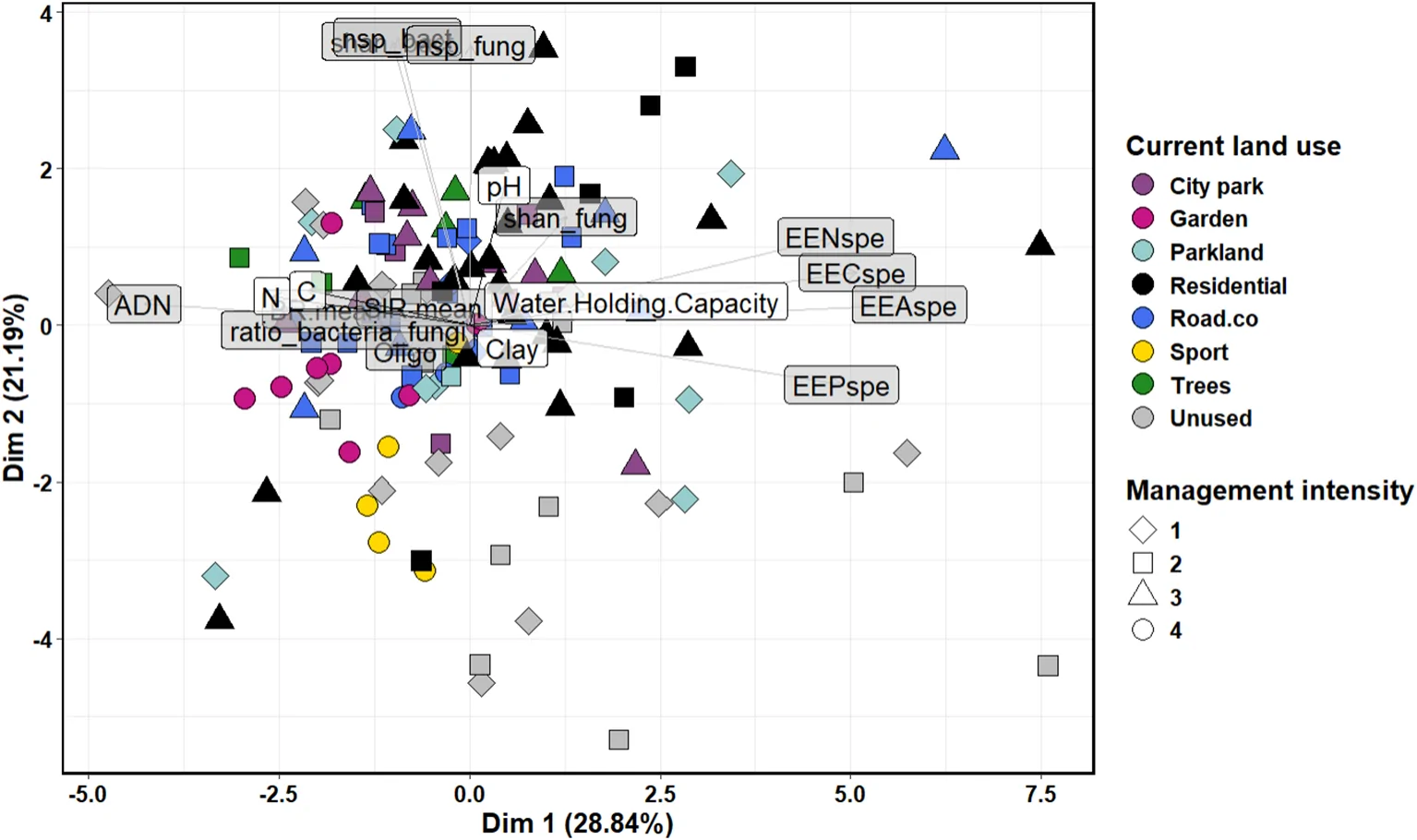
Principal component analysis (PCA) of functional indicators (enzyme analysis) and microbial parameters (DNA biomass, diversity and respiration). Colors of each point represent the current land use, while shapes indicate management intensity. Grey boxes denote the variables used for the PCA, whereas the white box represents supplementary soil variables.

## 4 Discussion

The present study investigated a data set of 135 soils under different land uses and management intensities to address several hypotheses that reflect the idea that microbial life strategies could serve as functional indicators of how urban land use and management intensity filter soil communities and reshape ecosystem processes. Through this work, we sought to enhance our knowledge of urban soils for which microbial communities are relatively uncharacterized in comparison to other environments, bringing new knowledge on the taxonomic and functional characterization of urban soil microbiomes which has recently been highlighted as a key priority for future research (Nugent and Allison, 2022).

### 4.1 Urban soils as a distinct microbial habitat: setting the context

Urban green spaces are shaped by anthropogenic factors such as pollution, soil compaction, mowing, fertilization and the introduction of non-native species. These practices contribute to variability in soil structure, nutrient availability and the diversity, richness and composition of microbial communities. Although previous studies have examined microbial diversity across broad climatic gradients, urban soils remain underrepresented in global microbial ecology research, despite their unique disturbance regimes and management practices

Bacterial richness averaged just above 1000 ASVs per sample, a range commonly reported in urban soils. Although some studies have found higher richness in cities across Australia, Cyprus, or France (Baruch et al., 2021; Stephanou et al., 2021; Christel et al., 2023), such comparisons are challenging due to differences in sampling methods, sequencing depth, and environmental contexts. At a French scale, our richness values are slightly lower than the national average (Terrat et al., 2017). In terms of community composition, the present study aligned with previous reports for French (Karimi et al., 2018) and other urban soils (Baruch et al., 2021; Stephanou et al., 2021; Grierson et al., 2023), with Proteobacteria as the dominant phylum, followed by Actinobacteria and Bacteroidota. Land use effects on bacterial composition have been reported for several phyla, including Acidobacteriota, Planctomycetota and Proteobacteria, and our results align with these patterns (Baruch et al., 2021; Stephanou et al., 2021; Christel et al., 2023; Grierson et al., 2023). Our study also revealed a diverse range of phyla, including Actinobacteria, Bacteroidota, Chloroflexi, Entotheonellaeota, Methylomirabilota, Myxococcota, NB1-j, Nitrospirota, and Planctomycetota, and Verrucomicrobiota, consistent with recent urban surveys.

Fungal richness averaged around 450 ASVs per sample, falling within ranges reported in other urban soil studies. Our values were lower than those observed in another French city (Christel et al., 2023) but were higher than those from an Australian urban context (Baruch et al., 2021), reflecting variability in fungal communities across urban environments. Fungal community composition, which has been less frequently characterized in urban soils, was dominated by Ascomycota followed by Basidiomycota, consistent with previous findings (Baruch et al., 2020; Grierson et al., 2023). Mortierella was also among the abundant taxa observed. Unlike bacterial communities, fungal composition showed few differences across land uses, except for some rare taxa. This pattern may partly reflect the choice of fungal marker: the broad ITS2 region maximizes taxonomic coverage but provides lower phylogenetic resolution potentially limiting the detection of fine-scale compositional shifts across land-use types.

Compared to natural ecosystems such as forests, grasslands, and shrublands, urban soils in our study exhibited lower network parameters indicating reduced microbial networks and weakened interaction strengths (Zheng et al., 2024). This fragility is likely a consequence of frequent anthropogenic disturbances.

### 4.2 Management intensity as an additional selective filter beyond land use

To isolate the effects of management intensity from land use, we defined a four-level management intensity ranging from 1 (lowest) to 4 (highest). Microbial richness and diversity showed a clear response: both fungal and bacterial richness peaked at the intermediate management intensity (level 3), consistent with the intermediate disturbance hypothesis and previous observations in urban soils (Baruch et al., 2021; Shen et al., 2022; Mawarda et al., 2025). At the highest intensity, richness and bacterial Shannon diversity declined. This may reflect soil acidification and nutrient imbalances associated with intensive nitrogen inputs, as previously reported (Zhang et al., 2018). This contrasts with other urban studies reporting higher microbial diversity in poorly managed or unmanaged soils (Stephanou et al., 2021; Grierson et al., 2023).

These divergent patterns diverged from studies showing higher microbial diversity in poorly managed or unmanaged soils, highlight that responses to management intensity may vary. The range of Road.co management intensities (from level 1 to 4) was associated with a wide variability, which may be attributed to the heterogeneity of urban environments. Road.co soils face additional pressures (pollution, trampling, and compaction) that were not directly measured here but have been previously identified as key drivers of soil properties and microbial composition (Greinert, 2015).

Management intensity also influenced microbial association patterns. In our study, bacterial and fungal communities in least managed areas (Unused areas) were more interconnected, with stronger co-occurrence patterns than in intensively managed areas. These co-occurrence networks do not provide direct evidence of ecological interactions or resilience, but they may indicate shifts in community organization consistent with stronger filtering under intensive management (Trivedi et al., 2017; Mandakovic et al., 2018).

These results complement those of Mawarda et al. (2025), who found that network complexity increased in more urbanized areas, suggesting that urban pressures may promote tighter niche specialization. This niche specialization could induce a reduction in network connectivity by preferentially associating some taxa together and reducing the networks’ overall strength. Indeed, we found stronger microbial associations in less-disturbed soils that may enhance essential ecosystem functions. In contrast, disrupted microbial networks in highly managed and Road.co soils may impair these functions, reducing soil ecological resilience (de Vries et al., 2018).

Although management intensity remains a relatively new metric in urban ecology, our results show that it significantly shapes microbial diversity, composition and association patterns. Refining and standardizing this indicator could improve future modeling of urban soil dynamics and support city-scale planning and green-space management.

### 4.3 Microbial richness, diversity and network complexity along the management gradient and land uses

Urban-specific disturbances create unique selection pressures that shape microbial communities by creating heterogeneous microhabitats across the city, leading to high variability in microbial communities. These disturbances vary strongly with land use. In our study, for example, only Garden and Sports areas received fertilization inputs and fungicide application was limited to Sports areas.

Bacterial richness varied across land uses, aligning with previous studies of urban soils (Baruch et al., 2020; Grierson et al., 2023). Bacterial diversity was highest in Residential and lowest in Unused and Sports areas. This reduced diversity in less managed areas may reflect a loss of microbial functions or a more specialized microbial community (Maron et al., 2011). Similarly, bacterial richness was lower in Unused areas compared to City park, Residential and Road.co areas. In contrast, Residential areas likely support more diverse and richer microbial communities potentially due to varied inputs like pollutants, pet waste, wastewater and organic debris.

Fungal richness varied across urban land uses, with patterns largely reflecting management intensity and organic matter availability. In our study, fungal richness was lower in Sports compared to Road.co areas and fungal diversity was the lowest in Sports compared to Unused areas, potentially due to fungicide application, which may selectively suppress non-targeted fungi. Indeed, the use of fungicide and manure practices can increase pathogen prevalence but decrease the overall diversity (Ma et al., 2021; Idbella and Bonanomi, 2023).

Fungal and bacterial beta-diversity revealed clear differences across land uses, clustering them into four distinct categories: Trees, Gardens, Sports and Unused. These reflect contrasting management intensities—from minimal intervention in Unused and Trees to intensive care in Gardens and Sports areas. Tree areas may foster unique microbial communities through root interactions and microclimatic effects (Labouyrie et al., 2023). Comparing these results with other studies of French urban soils is challenging due to differences in land use types and management. However, Christel et al. (2023) offers a good benchmark: they found that microbial composition varied across land uses, with public leisure areas showing lower diversity but higher biomass than traffic-related sites—a pattern partly mirrored in our Garden samples. Interestingly, fungal communities did not follow this trend, suggesting that similar land use labels can mask ecological differences. One notable difference concerns bacterial richness. Whereas Christel et al., observed lower bacterial richness in leisure soils, we did not observe such a pattern in our dataset: bacterial richness in Gardens did not differ significantly from the other land use types. These contrasting results highlight the complexity of urban ecosystems and the importance of continued research into how land use shapes soil microbial communities.

### 4.4 Shifts in microbial life-history strategies along the management gradient

Bacterial life strategies play a key role in community distribution across land uses (Piton et al., 2023). Oligotrophic bacteria—adapted to low-nutrient environments—were more abundant in Unused than in other land uses, except for the Garden. These slow-growing bacteria are known for their resilience in harsh conditions (Fierer, 2017; Trivedi et al., 2018; Malik et al., 2020), including urban soils (Baruch et al., 2021; Christel et al., 2023; Grierson et al., 2023). Their presence in both Unused and Garden areas may reflect distinct but converging ecological constraints: low nutrient availability in Unused soils and recurrent disturbance or resource fluctuations in Gardens, both of which can favor stress-tolerant oligotrophic taxa.

More precisely, Residential areas were dominated by copiotrophic Proteobacteria and Verrucomicrobiota, known to degrade complex carbon compounds (Baruch et al., 2021). This suggests that Residential soils host microbial communities adapted to organic matter inputs such as pollutants, animal waste and runoff. The possible influence of these factors is consistent with the reduced abundance observed for Chloroflexi which, although tolerant to pollutants, are generally adapted to oligotrophic, nutrient-poor conditions (Ma et al., 2022).

Unused areas supported distinct microbial communities, with elevated levels of Planctomycetota and Actinobacteria, and reduced Bacteroidota. *Planctomycetota*, often associated with polluted or wastewater-affected environments, are typically suppressed by fertilization and urban disturbance (Stephanou et al., 2021; Scholier et al., 2023; Garcia et al., 2024; Godinho et al., 2024; Koottatep et al., 2024). Their higher abundance in both Unused and Garden soil suggests a complex interplay of factors, including low management intensity, low nitrogen levels, and low carbon availability (Xu et al., 2024). Similarly, genera such as *Solirubrobacter* thrived in Unused areas, consistent with their preference for nutrient-limited conditions (Tan et al., 2024). While Actinobacteria are known for their adaptability and presence in diverse environments, they are not specifically classified as oligotrophic (Qin et al., 2019). Bacteroidota are copiotrophic bacteria that respond positively to organic amendments (Stone et al., 2023; Wu et al., 2024), explaining their lower abundance in the less-managed Unused areas. Garden showed the highest relative abundance of Nitrososphaeraceae a group often stimulated by nitrogen enrichment (Han et al., 2018).

This study provides a detailed and statistically supported analysis of fungal guild variations across urban land uses in which we observed a global trend of saprotrophic fungi being the most abundant guild (Baruch et al., 2020; Grierson et al., 2023). However, contrary to expectations based on previous nitrogen fertilization studies (Paungfoo-Lonhienne et al., 2015), saprotrophs were not more abundant in fertilized areas. Instead, their proportion was higher in less managed areas, likely reflecting the potential greater availability of undecomposed organic materials.

This information is also consistent with the Gadgil effect, where fertilization suppresses saprotrophs in favor of ectomycorrhizal fungi due to altered nutrient competition and shifts in decomposition pathways (Gadgil and Gadgil, 1971; Fernandez and Kennedy, 2016). Further analysis confirmed that trend where saprotrophic fungi were influenced by soil nitrogen content and pH, two key factors known to regulate fungal guild dynamics (Chen et al., 2019a).

Unexpectedly, mycorrhizal fungi were not more abundant in Trees areas compared to other land uses. This pattern appears to be driven not by land use itself, but by the identity and mycorrhizal type of the dominant tree species at each site. Our sampling sites included diverse tree genera such as *Quercus*, *Tilia*, *Fraxinus*, *Acer* (Maple), *Platanus*, *Pinus*, *Carpinus*, *Abies* (Fir), and *Alnus*, which differ in their preferred fungal partners—either arbuscular mycorrhizal (AM) or ectomycorrhizal (ECM). Thus, host-tree identity and symbiotic association type emerged as stronger determinants of mycorrhizal fungal abundance than land use or soil properties (Heděnec et al., 2023; Khalfallah, 2024; Khalfallah et al., 2024), which has important implications for urban green-space managers.

Beyond community-level patterns, our results suggest functional implications of dominant taxa. For example, copiotrophic Proteobacteria, abundant in Residential soils, are known to accelerate carbon turnover through rapid growth and high enzymatic activities, particularly β-glucosidase and aminopeptidase. The analysis of mediation confirms this trend where the Proteobacteria significantly partially mediate the effect of the β-glucosidase and other enzymatic activities on the soil carbon content and nitrogen content (**Supplementary Table S8**) for the urban soil. Conversely, oligotrophic Actinobacteria, prevalent in Unused areas, are associated with the decomposition of recalcitrant organic matter, supporting higher carbon-acquisition enzyme ratios (EEC: EEN). Similarly, fungal saprotrophs, dominant in less-managed soils, likely drive elevated carbon-cycle enzyme activities, whereas mycorrhizal fungi in Trees areas contribute to phosphorus acquisition, reflected in lower EEN: EEP ratios. However, the mediate analysis, demonstrate that root endophytes emerged as the main fungal mediators linking carbon- and nitrogen-cycle enzyme activities to soil C and N contents, indicating that part of the functional variation in less-managed soils operates through shifts in endophytic guilds (**Supplementary Table S9**).

### 4.5 Soil properties, limitations and cautions

While land use and management intensity were important, they were not the only drivers of microbial community structure. Our results highlight that soil properties, particularly carbon content, nitrogen availability and pH, were primary determinants of microbial richness, diversity, and composition. This suggests that biogeochemical conditions may have consistent effects across land use types, potentially due to shared soil histories or legacy effects from past management. Indeed, historical land use is known to exert lasting impacts on microbial communities (Dupouey et al., 2002; Fraterrigo et al., 2005; Khalfallah et al., 2024; Mejía et al., 2024). In Blois, a relatively young urban area, some sampling sites could retain characteristics of previous land use, such as former agricultural plots or undeveloped green spaces. Indeed, in this city the evolution of each land use and sampling site is variable for both the construction date and past land use (Deparis et al, 2025). These variations in site age and land use legacy likely contributed to long-term shifts in microbial communities observed in the present study.

A further limitation arises from the seasonal nature of our sampling. Because all soils were collected during a single season, our dataset does not capture temporal fluctuations in microbial communities driven by temperature, moisture, plant phenology or seasonal management practices. Urban soils can exhibit pronounced seasonal dynamics, and the absence of mult-season sampling may have constrained our ability to detect certain land use effects or to generalize patterns of microbial functioning across the year. Future work incorporating repeated seasonal sampling would help clarify how temporal variability interacts with land use, soil chemistry and management intensity to shape microbial communities.

Our data showed that urban soil bacterial richness was most strongly influenced by soil carbon content, followed by nitrogen, while bacterial diversity was primarily shaped by soil pH. In contrast, land use and management intensity had only minor effects on these bacterial parameters. These results further emphasize the fundamental role of soil physicochemical properties in structuring urban microbial communities. In our previous work, we also showed that land use strongly influences nitrogen-cycling processes, largely through shifts in microbial community composition and the distribution of key functional groups involved in nitrification and denitrification (Khalfallah et al., 2026). The relatively limited variation in microbial diversity across land uses, raises questions about the spatial scale of analysis. It is plausible that microbial communities respond more strongly to broader environmental gradients than to fine-scaled land use classifications. This lack of differentiation may explain the dominance of soil chemistry over land use type in influencing microbial dynamics.

Our classification did not include certain underrepresented land use categories, such as private gardens, bare soils, or artificial (engineered) soils. These types of urban spaces, which are often subjected to highly variable human management practices, could add significant complexity to our understanding of microbial diversity and function in urban environments. Their inclusion in future studies may help capture a more complete and more nuanced picture of urban soil microbial ecology.

Differences in microbial co-occurrence networks across land uses likely reflect variations in soil properties and microbial functioning. Garden and Sports areas consistently showed distinct microbial profiles, whereas Road.co, City Park and Trees displayed more variable responses. The variability in outcomes for certain land uses may stem from inconsistent definitions of management intensity, ranging from minimal intervention (e.g., City Park) to intensive disturbance (e.g., Road.co), but also from other disturbances such as the past land use that could lead to high variability between samples. These factors introduce substantial heterogeneity in soil parameters (pH, nutrient availability, organic matter content), which can obscure clear land-use signals and limit our ability to draw stronger conclusions.

Microbial co-occurrence networks also require careful interpretation. Network structure is highly sensitive to methodological choices, including filtering thresholds, normalization, and the statistical metrics used to infer associations. As a result, differences in network connectivity or modularity across land uses should be viewed as indicators of potential shifts in community organization, not as direct evidence of ecological interactions or resilience. Comparisons between our urban networks and those reported in natural ecosystems must also be made cautiously, as published networks often rely on different analytical pipelines, sequencing depths and taxonomic resolutions. Lower connectivity or more fragmented patterns in intensively managed soils may suggest reduced functional redundancy or altered niche structure, but these patterns should be interpreted with caution given the inherent sensitivity of network analyses.

## 5 Conclusion

Urban soil microbial communities are shaped by a powerful combination of land-use practices, management intensity and soil chemistry. Across the city, disturbances such as pollution, compaction, mowing and fertilization created contrasting soil environments and pH, nitrogen and carbon contents consistently emerged as the strongest drivers of microbial composition. Intensively managed sites stood out with elevated nutrient levels linked to repeated fertilization, clearly separating them from less-managed soils.

Land use acted as a decisive ecological filter. Sports showed the lowest microbial richness, diversity and functional activity, reflecting heavy disturbances. In contrast, Unused soils, despite their low nutrient status, supported highly interconnected communities dominated by oligotrophs and exhibiting strong carbon-acquisition enzyme activities. Garden combined high nitrogen with low enzyme activity, suggesting fertilization-driven shifts toward oligotrophic groups. Residential hosted diverse and functionally active communities, likely fueled by heterogeneous organic inputs and moderate disturbance, while Parkland displayed high soil and microbial values but lower diversity, pointing to nutrient-specialist assemblages.

These patterns revealed the complexity of microbial responses to urban pressures. Urban soils are shaped by multiple, often unpredictable factors—legacy effects, pollutant inputs, heterogeneous maintenance—that blur simple land use contrasts and highlight the dominant role of soil chemistry (Deparis et al., 2025). Understanding how microbial communities contribute to nutrient cycling, organic matter decomposition and soil resilience therefore requires moving beyond taxonomic inventories toward functional and interaction-based approaches.

Although the influence of land use and management intensity on microbial associations has long been hypothesized, empirical demonstrations remain scarce. By integrating diversity metrics, functional traits, enzymatic activities and co-occurrence patterns across a clearly defined management gradient, our study provides new evidence that management intensity acts as a key ecological filter structuring microbial communities in urban soils.

## Data Availability

The datasets presented in this study can be found in online repositories. The names of the repository/repositories and accession number(s) can be found below: https://doi.org/10.48579/PRO/MTZ0KB, PRJEB52222; https://doi.org/10.48579/PRO/MTZ0KB, PRJEB52287.
