# Supplementary material for: Urban land use influences microbial functional traits along a management intensity gradient
Source: Front Microbiomes. 2026 Sep 30;5:1946255. doi: 10.3389/frmbi.2026.1946255 (PMC13662681; doi:10.3389/frmbi.2026.1946255)
Supplement: Supplementary file 1 [file SupplementaryFile1.docx]

# Supplementary

**Supplementary Results**

**Tables in Supplementary File 2 Excel Document**

Table S1: Richness and diversity indices of soil fungi and bacteria in the different urban land uses. Letters indicate statistical differences performed with Tukey’s test (P<0.05).

Table S2: Richness and diversity indices of soil fungi and bacteria by land management intensity. Letters indicate statistical differences performed with Tukey’s test (P<0.05).

Table S3: Soil properties according to land use. Letters indicate statistical difference performs with Tukey’s test (P<0.05).

Table S4: Environmental predictor variables predicting soil microbial community composition with associated *envfit* R2 and Bonferroni-adjusted p-values for bacterial and fungal urban land uses from NMDS

Table S5: Bacterial and fungal relative abundances compared with different urban land uses. Letters indicate statistical difference performs with Tukey’s test (P<0.05).

Table S6: Bacterial and fungal relative abundances as a function of land management intensity. Letters indicate statistical difference performs with Tukey’s test (P<0.05).

Table S7: Significant abundances of bacterial genera from the most abundant ASV across different urban land uses. Letters indicate statistically significant differences based on Tukey's test (P < 0.05).

Table S8. Mediation analysis – bacteria assessing indirect and direct effects of bacterial taxa on soil carbon and nitrogen contents (ACME, ADE, total effects and proportion mediated).

Table S9. Mediation analysis – fungi evaluating indirect and direct contributions of fungal guilds to soil carbon and nitrogen contents, including ACME, ADE and proportion mediated.

Table S10.Network properties of bacterial and fungal co‑occurrence networks, including node connectivity, modularity, clustering coefficient and other topological metrics across land‑use categories.

Table S11: Environmental predictor variables predicting soil microbial composition parameters with associated *envfit* R2 and Bonferroni-adjusted p-values

**Tables in Supplementary File 3 Excel Document**

Table SM1: Land‑use × management intensity occurrence matrix. Counts of sampling points per combination of land‑use category and management‑intensity level.

Table SM2: Trait and lifestyle assignments with supporting references. List of bacterial and fungal trait/lifestyle classifications (copiotroph/oligotroph; fungal guilds), including taxonomic rank used and bibliographic sources.

Table SM3: Copiotroph vs. oligotroph proportions across land uses. Relative abundance (%) of copiotrophic and oligotrophic bacterial groups for each land‑use category.

Table SM4: Bacterial ASV‑level assignments. Life‑strategy classification (copiotroph/oligotroph) for each bacterial ASV, including taxonomic rank and confidence level.

Table SM5 :Fungal guild proportions. Relative abundance (%) of major fungal ecological guilds (saprotrophs, mycorrhizal fungi, endophytes, pathogens) across land‑use categories.

Table SM6 :Fungal ASV‑level guild assignments

**Supplementary Methods: See Supplementary File 4**

**Supplementary Figures**

**Figure S1**: PCoA plots of Roadside samples for (A) bacterial communities and (B) fungal communities. Colours and shapes indicate the level of land management intensity. Letters denote statistically significant differences in the positioning of points (Tukey test, P < 0.05).

**Figure S2:** Relative abundances of the oligotrophic bacteria as a function of the current land use (A) and the land management intensity (B). Tukey test (*=p-value <0.05, **=p-value <0.01, ***=p-value <0.001, ns=non-significant)

**Figure S3:** Top 15 most abundant bacterial taxa (16S rRNA). Colours indicate taxonomic affiliation at the phylum level, and bars represent mean relative abundance across land‑use categories. Suffixes denote taxonomic rank (e.g., o_ = order, f_ = family, g_ = genus, …)

**Figure S4:** Variance partitioning of microbial richness, diversity and of functional groups relative abundance (Oligotrophic bacteria) and fungal guild (Saprotrophic fungi, plant pathogen and mycorrhizal fungi) as a function of soil physicochemical (C, N and pH), current land use, management intensity and microbial tree trait.
