# Supplementary material for: Urban land use influences microbial functional traits along a management intensity gradient
Source: Front Microbiomes. 2026 Sep 30;5:1946255. doi: 10.3389/frmbi.2026.1946255 (PMC13662681; doi:10.3389/frmbi.2026.1946255)
Supplement: Supplementary file 2 [file SupplementaryFile4.docx]

**Supplementary Materials and Methods**

## **Bioinformatics analysis**

Raw 16S rRNA (V4–V5) and ITS amplicon sequences were generated on an Illumina MiSeq platform (GeT‑PlaGe, Auzeville, France). Bioinformatic processing was performed on the Galaxy platform using the FROGS workflow (Escudié et al., 2018). After sequencing, FASTQ files were demultiplexed according to sample barcodes. Quality filtering removed low‑quality reads, sequences containing ambiguous bases, and reads outside the expected amplicon length range (16S: 17 437–28 215 bp; ITS: 39 079–59 702 bp). Paired‑end reads were then merged with VSEARCH.

High‑resolution clustering was then carried out using the SWARM algorithm with an aggregation distance of d = 1, generating 1 029 377 initial prokaryotic ASVs and 348 614 initial fungal OTUs prior to chimera and abundance filtering. Chimeras were detected with VSEARCH and removed, eliminating 556 107 prokaryotic clusters and 57 739 fungal clusters, representing 22.4% and 2% of total reads, respectively. Low‑abundance features were also discarded using a minimum relative abundance threshold of 0.005% of total reads, leaving 2 413 sequences prokaryotic ASVs and 1 728 fungal OTUs. For fungal sequences, an additional ITSx filtering step was applied to remove non‑ITS regions, which eliminated 50 low‑quality ITS features, yielding the final curated ITS dataset used for downstream analyses.

Taxonomic assignment of prokaryotic ASVs was performed by BLAST against the SILVA 138.1 database (Quast et al., 2013), using a minimum identity of 97% and minimum coverage of 99%. Archaeal ASVs, including representatives of Crenarchaeota, were retained and included in all prokaryotic community analyses. In total, 2 409 prokaryotic ASVs were successfully assigned at least to the phylum level, and 1 149 to the genus level. Fungal OTUs were assigned using the UNITE Fungi 8.3 database (Abarenkov et al., [2010](https://besjournals.onlinelibrary.wiley.com/doi/full/10.1111/1365-2435.70302#fec70302-bib-0001)), with the same identity and coverage thresholds, resulting in 1 679 classified OTUs, of which 907 were resolved at genus level and 578 at species level.

To harmonize taxonomic resolution across markers and reduce redundancy, a second clustering step grouped SWARM units sharing ≥97% similarity and ≥99% identity.

**Sequencing depth and rarefaction.**

Sequencing depth varied moderately across samples, and rarefaction thresholds were selected based on the distribution of read counts to maximize sample retention while ensuring adequate coverage. All samples exceeded the rarefaction thresholds (23 669 reads for prokaryotes; 5 046 reads for fungi), and no samples were removed for insufficient depth. The fraction of reads discarded during rarefaction was moderate (43% for prokaryotes; 38% for fungi), and rarefaction curves confirmed that both datasets reached asymptotic coverage (Supplementary Fig. S1–S2), supporting the adequacy of sequencing depth for diversity analyses.

After normalization, the dataset contained 3 195 315 total prokaryotic reads (23 669 reads per sample; 2 413 ASVs) and 681 210 eukaryotic reads (5 046 reads per sample; 1 679 OTUs). Archaeal ASVs (e.g., Crenarchaeota) were retained and included in all analyses of prokaryotic community composition.

Figure 1. Rarefaction curves of 18S amplicon sequences showing sequencing depth and sampling coverage across all samples.

Figure 2. Rarefaction curves of 16S amplicon sequences showing sequencing depth and sampling coverage across all samples.

**Comprehensive Statistical Workflow**

All statistical analyses followed a unified workflow designed to ensure consistency across alpha‑diversity metrics, qPCR gene‑copy abundances, extracellular enzyme activities, relative‑abundance matrices, and co‑occurrence network analyses. Before any modelling, each dataset was examined to verify that the assumptions required for parametric or semi‑parametric analyses were met. Normality was assessed through visual inspection of histograms and Q–Q plots, and homogeneity of variances across land‑use and management categories was evaluated using standard tests of variance equality. Residuals from fitted models were systematically inspected to confirm that they were approximately normally distributed, exhibited constant variance, and did not contain influential observations. Cook’s distance and standardized residuals were used to identify potential outliers or leverage points. When variables displayed skewness or heteroscedasticity, appropriate transformations were applied to stabilize variance and improve model fit. Gene‑copy abundances and enzyme activities were log‑transformed, diversity indices were transformed using square‑root or logarithmic functions when needed, and relative‑abundance matrices were subjected to Hellinger transformation to reduce the influence of highly dominant taxa.

Alpha‑diversity metrics, including observed richness and Shannon diversity, were analysed using linear or generalized linear models depending on the distributional characteristics of the transformed data. Land‑use type was treated as a fixed factor, and management intensity was incorporated as a nested factor within land‑use to reflect the unbalanced sampling design. After model fitting, residual diagnostics were performed to verify normality, homoscedasticity, and independence. Pairwise comparisons among land‑use categories and management intensities were conducted using Tukey’s HSD. When several diversity metrics were analysed simultaneously, p‑values were adjusted using Holm or false‑discovery‑rate (FDR) correction to control for inflation of type‑I error. Effect sizes, expressed as η² or R², were reported to quantify the magnitude of the observed effects.

Gene‑copy abundances obtained by qPCR were analysed following the same modelling structure. After log transformation, models were fitted with land‑use and nested management intensity as fixed effects. Residual diagnostics were again used to confirm the validity of model assumptions. Post‑hoc comparisons were performed using Tukey’s HSD, and p‑values were adjusted using Holm or FDR correction when multiple qPCR targets were evaluated. Effect sizes were reported to facilitate interpretation of ecological relevance.

Extracellular enzyme activities and derived ratios were analysed using linear or generalized linear models after log transformation. Composite indices representing carbon‑, nitrogen‑, and phosphorus‑related enzyme activities were included in the analysis, and ratios such as EEC:EEN and EEN:EEP were transformed when necessary to meet model assumptions. Because enzyme datasets involve multiple response variables, p‑values were systematically corrected using FDR procedures to limit false positives. Effect sizes were reported for all enzyme‑related models.

Microbial community composition was analysed using Hellinger‑transformed relative‑abundance matrices. Bray–Curtis dissimilarities were computed and used as the basis for all beta‑diversity analyses. Differences in community composition were assessed using PERMANOVA with 999 permutations, with permutations constrained within land‑use categories to respect the nested design. Homogeneity of multivariate dispersion was evaluated using betadisper followed by permutation tests, and PERMANOVA results were interpreted only when dispersion was homogeneous. Ordination analyses were performed using NMDS and PCoA. NMDS stress values were examined to ensure adequate representation of multivariate structure, and ordination ellipses were interpreted as descriptive data ellipses rather than confidence intervals. Environmental variables, including soil physicochemical properties, enzyme activities, respiration rates, and gene‑copy abundances, were fitted onto ordinations using envfit with 999 permutations. Because envfit evaluates each variable independently, p‑values were adjusted using FDR correction when multiple soil variables were tested simultaneously.

Co‑occurrence network analyses were conducted using CLR‑transformed ASV abundance data, Pearson correlations to estimate associations, and pseudo‑count zero handling. To ensure comparability across land‑use categories, a minimum ASV richness threshold was applied, corresponding to the lowest richness observed among samples (737 ASVs for bacteria and 206 ASVs for fungi). A minimum sequencing‑depth threshold was also applied, matching the rarefaction depth used in community analyses (5040 reads for bacteria and 23,669 reads for fungi). Only samples meeting both criteria were retained for network inference. Land‑use categories with insufficient sample sizes after filtering were grouped to maintain comparable sample sizes across networks. Community detection was performed using the fast‑greedy clustering algorithm, and network properties such as the size of the largest connected component, clustering coefficient, modularity, proportion of positive associations, edge density, natural connectivity, vertex and edge connectivity, average dissimilarity, and average path length were quantified. Network edges were interpreted strictly as statistical associations rather than ecological interactions, and no claims were made regarding robustness, resilience, or functional redundancy.

Across all analyses, multiple‑testing corrections were applied systematically. Tukey’s HSD controlled the family‑wise error rate for pairwise comparisons within models, while Holm or FDR correction was applied to sets of independent tests such as enzyme activities, soil variables, and environmental fitting. All results were reported with effect sizes, adjusted p‑values, and diagnostic checks to ensure that statistical assumptions were met. Interpretation was restricted to statistically supported patterns, avoiding causal claims where not justified.
